# A dose-finding design for dual-agent trials with patient-specific doses for one agent with application to an opiate detoxification trial

**DOI:** 10.1002/pst.2181

**Published:** 2021-12-10

**Authors:** Pavel Mozgunov, Suzie Cro, Anne Lingford-Hughes, Louise M. Paterson, Thomas Jaki

**Affiliations:** 1 MRC Biostatistics Unit, University of Cambridge, Cambridge, UK; 2 Imperial Clinical Trials Unit, School of Public Health, Imperial College, London, UK; 3 Division of Psychiatry, Department of Brain Sciences, Imperial College, London, UK; 4 Department of Mathematics and Statistics, Lancaster University, Lancaster, UK

**Keywords:** baclofen, combination trial, dose individualisation, dose-finding, methadone, opiate detoxification

## Abstract

There is a growing interest in early phase dose-finding clinical trials studying combinations of several treatments. While the majority of dose finding designs for such setting were proposed for oncology trials, the corresponding designs are also essential in other therapeutic areas. Furthermore, there is increased recognition of recommending the patient-specific doses/combinations, rather than a single target one that would be recommended to all patients in later phases regardless of their characteristics. In this paper, we propose a dose-finding design for a dual-agent combination trial motivated by an opiate detoxification trial. The distinguishing feature of the trial is that the (continuous) dose of one compound is defined externally by the clinicians and is individual for every patient. The objective of the trial is to define the dosing function that for each patient would recommend the optimal dosage of the second compound. Via a simulation study, we have found that the proposed design results in high accuracy of individual dose recommendation and is robust to the model misspecification and assumptions on the distribution of externally defined doses.

## 1 Introduction

Increasingly clinical trials investigate the combination of several treatments as they are thought to have the potential of inducing better therapeutic effects.^1^ The objective of Phase I combination studies is to find the highest treatment dose combination associated with the maximum acceptable toxicity, referred to as the maximum tolerated combination. To support the increase in demand, a number of novel dose-finding designs for dual-agent combination trials have recently been proposed.^2–6^ Most of these methods focus on the setting where a discrete number of doses are to be explored for both agents and the application is primarily in cancer trials. In parallel, Phase I trial designs for dual-agents with continuous doses have also been developed.^7^

For combination treatments where one of the agents is given at a fixed level, Mozgunov et al.^8^ proposed a combination design where the backbone agent is fixed at the same level for all patients, while a discrete set of doses of another agent is escalated. Huo et al.^9^ used a similar setting initially except, that, after the maximum tolerated dose of the discrete-dose agent is found, the continuous dose of the backbone agent is adjusted. Both of these proposals concerned the application in cancer trials, and their distinguishing feature is that the dose of the backbone therapy administered is the same for all patients. However, there are often many potential factors that can define the dose of the backbone agent for individual patients, for example, age, weight, previous disease record, and so on, so that this dose is often patient-specific. Therefore, when studying combinations of the backbone agent and a new therapy, the patient-specific dose of the backbone agent should be accounted for when selecting the maximum tolerated dose of another agent.

Patient-specific dosing has become an area of recent investigation. Specifically, at least two dose-finding trial designs for single-agent studies that allow for the selection of the biomarkers and biomarker-specific dose recommendation were recently proposed.^10,11^ However, at the same time, the setting of patient-specific combination recommendation has received less attention in the literature to date.

This work is motivated by a clinical trial of opiate detoxification led by the Division of Psychiatry Imperial College, funded by the Medical Research Council, reference MR/T025557/1. The trial consists of two parts: Study 1 is a dose finding trial that aims to study the safety of baclofen in combination with methadone, followed by a randomized controlled proof-of-concept Study 2 to assess whether baclofen, can facilitate detoxification from methadone. We will focus on Study 1 which investigates dual-agent combinations of four doses of baclofen (10, 30, 60, 90 mg) and various continuous doses of methadone in opiate-dependent individuals. The distinguishing feature of this trial is that the dose of methadone is patient-specific and defined externally to the study as the dose prescribed by the treating clinician. This dose of methadone is defined prior to enrolling in the trial. Hence, the maximum tolerated dose of baclofen can vary for different individuals and may depend on the prescribed dose of methadone. Therefore, the objective of the trial is to define the *dosing function* that for each patient, given their individual dose of methadone, will recommend the dose of baclofen that is associated with 15%−25% risk of a dose-limiting toxicity (DLT) in them. This objective is different to the conventional single-agent or combination dose-finding studies targeting a single dose/combination.

Whilst only one compound in the combination is escalated, the trial cannot be regarded as a conventional singleagent study as the dose of methadone will (likely) influence the risk of DLT in the combination. This study could be considered as a single-agent dose-escalation trial with a patient-specific dose of methadone being a covariate, similar to the design proposed by Bailey et al.^12^ However, the original proposal considers discrete dose levels of standard of care only. While one can adopt the proposal to continuous doses, a natural question of how the drugs (and their interaction) should be included into the joint combination−toxicity model arises. Moreover, it might be of interest to interpolate the toxicity risk between the studied discrete doses of baclofen, and binary covariates will not allow for this.

In this work, we propose a Bayesian model-based dose-finding design for a dual-agent combination study, in which one of the compounds (given either in discrete or continuous doses) is determined externally for each patient individually and the other compound is escalated. The proposed design adapts the extension of the two-parameter Bayesian Logistic Regression Model (BLRM)^13^ to a dual-agent combination setting proposed by Neuenschwander et al.^14^ We demonstrate how the parameters of the proposed design can be calibrated for the given clinical setting, and illustrate how the operating characteristics of the proposed design can be comprehensively evaluated and clearly communicated to clinicians and funders using new predictive performance metrics and combination escalation/de-escalation paths. We compare the performance of the proposed design to the model by Bailey et al.^12^ with binary covariates.

The paper proceeds as follows. The design is proposed in Section 2. The behaviour of the design for two possible realisations of the trial and example of the output used to communicate the design’s decisions to the clinical team throughout the trial are given in Section 3. A numerical study, including calibration of the hyper-parameters, a comprehensive simulation study is given in Section 4. Section 5 provides a sensitivity analysis before Section 6 concludes with a discussion on the proposed application and flexibility of the approach in other clinical settings.

## 2 Methods

### 2.1 Setting

Let *M*∈[*M*_min_,*M*_max_] be the patient-specific dose (on a continuous scale) of the first compound, which is defined externally and *M*_min_,*M*_max_ are the minimum and maximum doses of the compound studied in the trial. Let *B*_1_,…,*B*_k_,…,*B*_K_ be *K* doses of the second compound which is escalated in the trial. Denote the probability of a DLT given doses of *M* and *B_k_* as monotherapies by *p*(*M*) and *p*(*B_k_*), respectively, the probability of a DLT given administering combinations *M* and *B_k_* together by *p*(*M,B_k_*) and the odds transformation of a probability *q* by odds $\left(q\right)=\frac{q}{1-q}.$ The objective of the trial is to study the safety of the combination of the compounds, and, specifically, to define the *dosing function* that, for each given dose of the first compound, *M*, will recommend the dose of the second compound, $B_{k}|M,$ that is associated with the risk of a DLT lying in the interval [*γ−δ,γ+δ*] where *γ* is the target probability and *δ* is the half-width of interval in which the dose is considered close enough to the target toxicity characteristics. The trial is sequential with *N* being the total numbers of participants, and the estimates of the dosing function being updated after each group of *c* patients. Note that each patient in the group receives their specific (and most likely different) dose of *M* as prescribed by an external physician, and dose of *B_k_* given the sequentially updated dosing function. The design described below can be used in settings with either continuous or discrete doses of agent *M* but we will continue to consider continuous doses as in the motivating trial.

### 2.2 Combination-toxicity model

The combination−toxicity relationship under the assumption of independence of the compounds can be written as

$$p_{0}\left(M,B_{k}\right)=1-\left(1-p\left(M\right)\right)\left(1-p\left(B_{k}\right)\right).$$

Neuenschwander et al.^14^ proposed to include the interaction term in the combination−toxicity model as follows

(1) $$\text{odds}\left(p\left(M,B_{k}\right)\right)=\text{odds}\left(p_{0}\left(M,B_{k}\right)\right)\times \exp\left(\eta \frac{M}{M_{*}}\frac{B_{k}}{B_{⁎}}\right),$$

where M_⁎_ and *B_⁎_* are reference doses, and *η* is the interaction coefficient, positive values of which correspond to synergistic toxicity, zero corresponds to additive effect, and negative values correspond to antagonistic toxicity. Consequently, the quantity to be defined is the probability of toxicity associated with each agent given as a monotherapy, *p*(*M*), *p*(*B_k_*). Note that *p*(*M*) models the population-average risk across all individuals with the methadone dose prescribed at *M*. Given the motivating trial, we require that these probabilities can be modelled for both discrete and continuous doses in the trial. Therefore, the dose-toxicity models for each agent given as monotherapies are parameterised by a two-parameter logistic log-normal model with the actual dosage of the agent included directly in the model on the log-scale^13^

$$\begin{array}{l} \text{logit}\left(p\left(M\right)\right)=\alpha_{01}+\alpha_{11}\times \log\left(M/M_{*}\right),\\ \text{logit}\left(p\left(B_{k}\right)\right)=\alpha_{02}+\alpha_{12}\times \log\left(B_{k}/B_{*}\right), \end{array}$$

with prior $\left(\alpha_{0i},\log\left(\alpha_{1i}\right)\right)∼N\left(\mu_{i},\sum_{i}\right)$ where $\mu_{i}={\left(\mu_{0i},\mu_{1i}\right)}^{\text{T}}$ is the vector of means and

$$\sum_{i}=\left[\begin{array}{cc} \sigma_{0i} & \sigma_{01,i}\\ \sigma_{01,i} & \sigma_{1i} \end{array}\right]$$

is the covariance matrix, and $\eta ∼𝒩\left(0,\sigma_{\eta }^{2}\right).$ The reference doses are selected for each trial setting individually and can correspond, for example, to those doses at which the toxicity risk is desired to be modelled directly (as *α*_11_ or *α*_12_ will not contribute to modelling their toxicity). Note that when *M* = 0 (or *B* = 0), the underlying model simplifies to the conventional two-parameter logistic model for a single-agent as $\log\left(M/M_{*}\right)=-\infty$ implies *p*(*M*)=0. This is one of the desirable model features as it also enables setting prior probabilities to the individual dose model parameters using external data directly, which might be available for some of the agents.^15^

Parameters *α*_0*i*_,*α*_1*i*_,*η* are the unknown quantities that define the combination−toxicity relationship. The posterior distribution of these are sequentially updated using a prior distribution and the data collected during the trial using Bayes theorem. Specifically, denote the joint prior distributions of vector *θ* = (*α*_01_,*α*_02_,*α*_11_,*α*_12_,*η*) by *f*_0_ (.). Assume that *n* patients have been observed and received combinations (*M*_(1)_,*B*_(1)_)…, (*M*_(*n*)_,*B*_(*n*)_) and binary responses $Y_{n}={\left[y_{\left(1\right)}\ldots ,y_{\left(n\right)}\right]}^{\text{T}}$ were observed, where subscript (*j*) corresponds to the doses of the respective treatment given to the *j* ^th^ recruited patient. The model updates the posterior distribution of *θ* as

(2) $$f_{n}\left(\theta \right)=\frac{f_{n-1}\left(\theta \right)\phi \left(M_{\left(n\right)},B_{\left(n\right)},y_{\left(n\right)},\theta \right)}{\int {\;}_{{\mathbb{R}}^{5}}f_{n-1}\left(u\right)\phi \left(M_{\left(n\right)},B_{\left(n\right)},y_{n},u\right)\text{d}u}=\frac{f_{0}\left(\theta \right)\prod_{i=1}^{n}\phi \left(M_{\left(i\right)},B_{\left(i\right)},y_{\left(i\right)},\theta \right)}{\int {\;}_{{\mathbb{R}}^{5}}f_{0}\left(u\right)\prod_{i=1}^{n}\phi \left(M_{\left(i\right)},B_{\left(i\right)},y_{\left(i\right)},u\right)\text{d}u}$$

where

$$\phi \left(M_{\left(n\right)},B_{\left(n\right)},y_{\left(n\right)},\theta \right)=p{\left(M_{\left(n\right)},B_{\left(n\right)},\theta \right)}^{y_{\left(n\right)}}{\left(1-p\left(M_{\left(n\right)},B_{\left(n\right)},\theta \right)\right)}^{1-y_{\left(n\right)}}.$$

This posterior distribution is then used to make escalation and de-escalation decisions for *B_k_* during the trial, for a given *M*.

### 2.3 Dose-escalation design

We begin by defining practical escalation restrictions that define the *admissible* set of combinations that originate from the motivating trial. Doses of the escalated compound *B* cannot be skipped regardless of the externally defined patient-specific dose of the compound *M*. At the same time, the doses of *B* do not have to have been given together with the dosages of *M* already tried in the trial in order to be escalated. Additionally, the coherency condition^16,17^ adopted for the combination setting needs be satisfied: if at least one of the patients in the previous group of patients experienced a DLT, the next group of patients cannot receive higher dose of the dose-escalated compound *B* than received by the individual(s) experiencing the DLT in the previous group regardless of the patient-specific externally defined dose of *M*. A dose of *B* satisfying this condition would be called an admissible dose. Note that these restrictions are specific to the motivating trial and could be relaxed for other applications if appropriate.

Then, the design takes the form

1. The fixed patient-individual dose of *M* and the lowest dose of *B_k_* are allocated to the first group of c patients.
2. After the DLTs are evaluated for the previous *c* patients, the posterior distribution of *α*_0*i*_,*α*_1*i*_,*η* is updated using equation (2) and the admissible doses of *B* are defined.
3. When the next patient comes into the trial, with a pre-determined dose of $M^{\prime }$ , assigned by the treating physician, the estimated set *of safe* admissible doses of baclofen (given the fixed dose of methadone) is found as
$$\text{ℙ}\left(p\left(M=M^{\prime },B_{k}\right)>\gamma +\delta \right)<c_{\text{overdose}}$$
  where *c*_overdose_ is the threshold controlling the risk of overdosing, and *γ* + *δ* is the risk above which the combination is considered unacceptably high.
4. Then, among the set of safe admissible doses, *A*, the dose of *B* that has the highest probability of having a risk of DLT between (*γ − δ*) % and (*γ* + *δ*) % given the dose *M^′^* is assigned to this patient
$${\text{argmax}}_{B_{k}\in A}\text{ℙ}\left(\left(M=M^{\prime },B_{k}\right)\in \left(\gamma -\delta ,\gamma +\delta \right)\right)$$
  where the probability is found with respect to the posterior distribution of *θ*.
5. Steps 2−4 are repeated until the maximum number of patients is reached or trial-specific early stopping criteria are met (see Section 3.2 for details).

The parameters *γ*,*δ*,*c*_overdose_ are trial specific and will be defined in the setting of the motivating trial below. As the optimal dose of *B* is patient-specific and is expected to depend on the externally prescribed dose of *M*, the output of the design (if the trial was not stopped earlier) is the estimated dosing function of the form (1) with parameters having posterior distribution as defined by (2) at the end of the trial. When provided with a dose of *M*, the output of this dosing function is the dose of *B* that is the most likely to be in the target toxicity range.

Examples of the design’s dose-escalation recommendations in individual trials together with the example output that can be provided to the clinical team are given in the next section.

## 3 Individual Trial Behaviour

### 3.1 Setting

In this section, we consider two examples of the dose-escalation in the setting of the motivating trial when using the proposed design.

The objective of the motivating trial is to define a dosing function that for a given dose of methadone will recommend the dose of baclofen that is associated with toxicity risk between 15% and 25% (*γ*= 0.20,*δ*= 0.05) for a given patient. The maximum sample size in the trial is *N* = 48 and the group size is *c* = 3. Generally, the estimates of the dosing function can be updated after each patient. However, the choice in favour of updating the model every three patients was made for logistic purposes and to collect more information about baclofen doses for various doses of methadone. We fix the overdosing constant *c*_overdose_ = 0.25 that was demonstrated to have good safeguarding properties for the five-parameter logistic model.^14^

### 3.2 Early stopping criteria

In discussion with clinicians, it might not be always desirable to proceed the trial until the maximum number of patients are recruited and, hence, early stopping is of interest.

Firstly, the clinical team of the trial advised that it would be strategically infeasible to proceed to the next phase of the study if a particular combination of methadone and baclofen are deemed to be unsafe. Specifically, the clinicians specified that the smallest clinically meaningful (to proceed to the next proof-of-efficacy trial) combination of 60 mg methadone and 30 mg baclofen being safe should be used as a criterion to proceed to the next proof-of-concept phase. We use this combination throughout the trial to check whether it is ethical to continue the trial. Formally, if

(3) ℙ(p(M=60mg,B=30mg)>0.25)>0.25,

the trial will be recommended to stop earlier for safety concerns. Triggering the safety constraint would mean that the subsequent use in clinical practice would be very limited.

Secondly, it was of interest to be able to stop the trial earlier if it was found that the highest doses of baclofen are safe even for high doses of methadone (which was chosen to be 120 mg by the clinical team). However, it was required that the trial is stopped earlier for this reason only if there is significant evidence of the high combination being safe. Formally, if

(4) ℙ(p(M=120mg,B=90mg)<0.25)>0.925,

then the trial could be stopped concluding that all baclofen doses are safe. The constant 0.925 was tuned via simulations (Section 4) to achieve early stopping in safe scenarios and avoid them otherwise. It means that only if we are at least 92.5% confident that the high combination is safe, the trial can be stopped earlier.

### 3.3 Prior parameters

The proposed design requires the hyper-parameters *μ*_0*i*_,*μ*_1*i*_,*σ*_0*i*_,*σ*_1_ *σ*_01,*i*_ (*i* = 1,2) and *σ_η_* and references doses M* and B* to be pre-specified before the start of the trial. The references doses were chosen to be directly linked to the combination at which the safety constraint is checked, *M*_⁎_= 60mg and *B*_⁎_= 30mg to simplify communicating the model to the team. The hyper-parameters were tuned via extensive simulations over a range of qualitative different scenarios—see further details on the hyper-parameter calibration in Section 4.4. It was found that *μ*_01_ = −3.75, *μ*_02_ = −3.25, *μ*_11_ = 0.40, *μ*_12_ = 0.05,*σ*_0*i*_=0.50,*σ*_1*i*_ =0.35,*σ*_01,*i*_=0.0,*σ*_*η*_=1.25 yield good properties of the design across scenarios with various combination-toxicity relationships.

**Figure 1:**
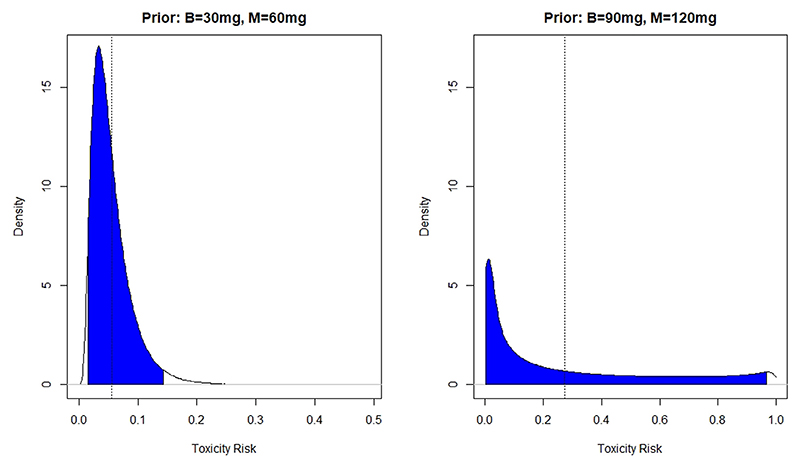
Prior distribution of the toxicity risks at combination (*M* = 60mg, *B* = 30mg), left panel, and combination (*M* = 120mg,*B* = 90mg), right panel, and corresponding 95% credible intervals. The mean of the distributions are marked by the dotted lines

The prior distribution on the model parameter implies a prior distribution on the toxicity risk at given combination. These prior distributions of risk can be more intuitive to interpret and communicate then the prior distribution on parameters. The prior distribution of toxicity risks at two combinations (*M* = 60mg,*B* = 30mg) and (*M* = 120mg,*B* = 90mg) that used in the early stopping criteria (3)−(4) and associated 95% credible intervals are given in Figure 1.

Considering the prior distribution for the lower combination, the hyper-parameters imply that it is safe with high probability that was in line with the clinicians’ expectations. At the same time, the prior distribution on the highest combination is noticeably more uncertainty with the 95% credible interval being almost over the whole unit interval. This, again, was in line with the clinicians’ knowledge as there is high uncertainty whether the highest combination of baclofen will be well tolerated with high dose of methadone.

Below, we consider two examples of how the escalation/de-escalation would look if (i) no DLTs are observed in the trial, and (ii) DLTs are observed in the trial. The design was implemented using R.^18^

### 3.4 Example 1

The first example is the escalation path when no DLTs are observed in the trial (Table 1).

Table 1 contains the externally prescribed methadone doses (in the group of three patients), baclofen doses recommended by the design, DLTs outcomes observed for the corresponding group of patients, ℙ(unsafe) and ℙ(safe), the probabilities used for early stopping criteria (3)-(4), and columns *B =* XXmg corresponding to the intervals of methadone that a patient should be prescribed in order to be recommended dose XXmg of baclofen given the DLTs outcomes observed for the current and previous groups. The dose of methadone is capped at 150 mg and starts at 10 mg (minimum eligible dose). The prescribed dose of methadone is randomly generated for the purpose of illustration with the distribution being informed by the recent data in the studied population (see Section 4 for details). In each row, the last columns are used to assign next group of patients (subject to the escalation constraints) given their methadone doses (M doses in the next line).

The trial starts at the lowest dose of baclofen, as required, and after no DLTs in the first group the dose of baclofen is escalated. At this point, the probability of the highest combination being safe is close to 50% compared to 92.5% required to terminate the trial earlier. After no DLTs are observed at the next dose, the dose of baclofen is escalated again until the highest dose of 90 mg is reached. Four groups in total are assigned to the highest combination before reaching the conclusion that all combinations are safe. One of the reasons for the trial not stopping earlier is the actual doses of methadone tried in the trial. Specifically, to reach the conclusion that the highest dose of baclofen is safe even for high doses of methadone, the corresponding high doses of methadone should be studied in the trial to avoid that these are inadequately extrapolated from lower doses by the selected model.

Note that as the trial progresses the probability of the highest combination being safe increases but the increase is smaller for low doses of baclofen and higher when the highest dose of baclofen (that is used in the early stopping criteria) is used. Similarly, the interval of methadone doses for which 90 mg of baclofen are recommended gradually widens as the trial progress but the change is more minor for lower doses of baclofen and becomes more prominent as the highest dose of baclofen is being assigned. At the same time, the intervals of methadone doses for which 10 and 30 mg of baclofen should be recommended narrow down and empty as more evidence of the combination being safe is gathered.

The outcome of the model is the recommendation that all doses of baclofen are safe when given with the dose of methadone up to 120 mg.

**Figure 2:**
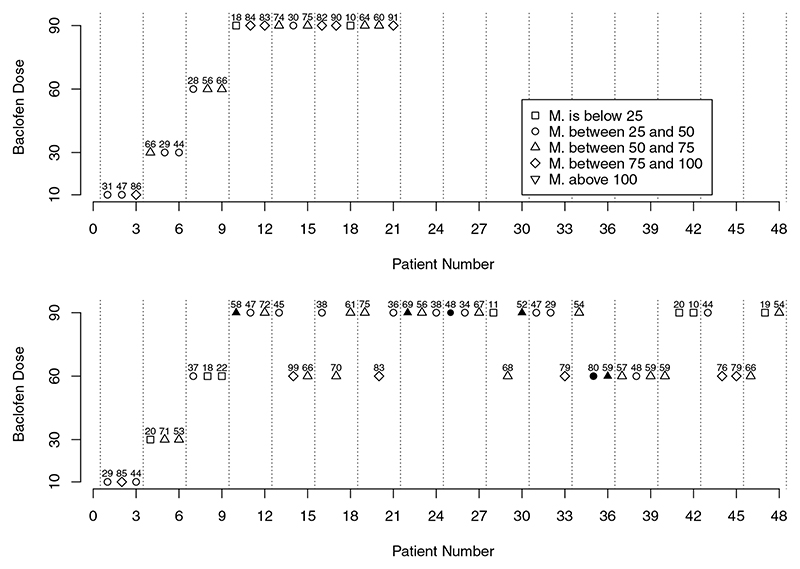
Two possible escalation/de-escalation paths in the individual trial. The patient number is on the *x*-axis, the dose of baclofen is on the *y*-axis and the dose of methadone (in mg) is above the corresponding allocation dose. The shape of the point corresponds to different intervals of the methadone doses, and the open symbol corresponds to no DLT, and a filled symbol corresponds to a DLT

Together with the table above, we have found that presenting the escalation path suggested by the design as a figure was a useful tool to communicate the decisions of the model to the clinical team. The figure provided to the clinicians for the considered escalation path is given in Figure 2 (top panel).

In these trajectories, the patient number is on the *x*-axis, the dose of baclofen is on the y-axis and the dose of methadone (in mg) is above the corresponding allocation dose. The shape of the point corresponds to different intervals of the methadone doses, and the open symbol corresponds to no DLT, and a filled symbol corresponds to a DLT. This was used an illustration to show how a hypothetical trial would continue were the recommendations of the model followed throughout the trial.

### 3.5 Example 2

The second example of an escalation path with DLTs being observed in the trial is given in Table 2. The format of the table is the same as in Example 1.

The second example (the part in bottom panel in Figure 2) corresponds to a trial that continues until all 48 patients are recruited and several DLTs are observed in the trial. Again, the trial starts at the lowest dose of baclofen and it is escalated to the highest dose as no DLTs are observed in the three first groups. The probability of the highest dose of baclofen being safe gradually increases at this point with the interval of methadone doses for which 90 mg of baclofen would be recommended marginally widening. When the highest dose is reached, one DLT in a patient who received 58 mg of methadone was observed. The probability of the highest dose of baclofen being safe drops immediately, and the intervals of methadone doses for which higher doses of baclofen should be recommended go down. As a result, the next group enrolled in the trial (group 4) received different recommended doses of baclofen. Specifically, for the two patients with methadone dose above 58 mg (for which the previous DLT was observed), the dose of baclofen is deescalated to 60 mg while for the patient with a methadone dose of 45 mg the baclofen dose is the same as for the previous group, at 90 mg. This is in line with the interval recommended after the analysis including the fourth group. As no DLTs are observed for this group, the probability of the highest dose being safe increases and the intervals of methadone for which corresponding doses of baclofen should be recommended go up. Note that the increase in these interval is to a smaller extent than a decrease when a DLT was observed in the previous group.

The same patterns are observed every time DLT(s) are observed during the trial. Further in the trial, patients with higher doses of methadone start to receive higher dose of baclofen but more gradually.

The output of the design by the end of the trial is, essentially, the last line in Table 2—the ranges of the methadone doses for which corresponding doses of baclofen should be recommended. Specifically, patients with prescribed dose of methadone between 10 and 62 mg would be recommended 90 mg baclofen; patients with methadone dose between 63 and 92 mg would be recommended 60 mg; with methadone dose between 93 and 142 mg—30 mg baclofen; and with methadone dose between 143 and 150 mg—10 mg baclofen. The probability that the highest dose is safe is very small and hence the assignment in the next phase should proceed with caution.

Overall, the provided combination-escalation paths were demonstrated to the clinicians and it was agreed that the design leads to intuitive decision-making during the trial in line with expectations concerning the mechanism of action of the considered compounds.

The results above represent only two possible scenarios, and it is of interest to study the properties of the proposed design on average over a range of scenarios. This is done via a simulation study below.

## 4 Simulation Study

### 4.1 Setting

In this section, we evaluate the performance of the proposed five-parameter logistic combination−toxicity model in a comprehensive simulation study using the setting of the motivating trial introduced in Section 3. We are interested in (i) how accurate the design recommendations are, (ii) how many patients are assigned in the trial, (iii) how many of them experience a DLT, and (iv) what proportion of trials are stopped before the maximum number of patients is reached.

While metrics (ii)−(iv) are conventional in dose-escalation trials, a possibility of each patient having a different optimal dose of baclofen makes the conventional accuracy metric, the proportion of the correct dose selections, not applicable in this setting. Therefore choosing an appropriate operating characteristic of the design accuracy that can be clearly communicated to the clinical team and a funder is challenging.

### 4.2 Accuracy performance metric

In general, there is a contour of the target combination − for each given dose of *M*, there is a target dose of the *B* compound, and one can report how well this contour is fitted across doses of *M* by the end of the trial. Communicating this approach to the clinical team or funder can, however, be challenging as the “closeness” of the estimated contour to the true one is not straightforwardly interpretable. Therefore, we propose to use two new measures of accuracy of the proposed design instead.

The output of the trial is not a single dose (or combination) recommendation but a dosing function answering the question: “For the given dose of *M*, which dose of *B* should be recommended to this individual patient?” As a successful dose-escalation Phase I is usually followed by an aligned Phase II, then this dosing function can be used to make recommendation for the patients in the subsequent phases. Therefore, we evaluate the performance of the design in terms of its predictive properties for the patients to be enrolled in the next phase.

Specifically, for the motivating trial, the plan is to follow this dose-escalation study with a proof-of-concept study in which 112 patients will be randomised between the target combination and the control. We will assess the accuracy of the proposed dose-finding design by the proportion of patients (out of these 112) that will be recommended (i) their target combination, and (ii) a combination that is safe for them. Then, the summary characteristics over a number of simulations will be the average proportion out of these 112 patients assigned to the target and safe combinations.

### 4.3 Scenarios

To demonstrate the design has good properties in various clinical settings, we consider several simulation scenarios based on different combination−toxicity relationships. As the doses of the *M* compound are patient-specific and determined externally to the trial, within the simulation study one needs to make an assumption for the doses of *M* that the up to 48 patients entering the trial will be receiving. For this, previous data on the methadone doses of patients eligible to take part in the trial (provided by the Division of Psychiatry, Imperial College from ALH’s work in addiction services at Central North West London NHS Foundation Trust, not yet publicly available) were used. Consequently, the doses of methadone are generated from the truncated normal distribution with the mean 51.4 mg and standard deviation of 23.3. The distribution is truncated at the minimum dose of methadone that would likely be the minimum clinically relevant dose for this trial, namely 10 mg. We study the robustness of the design to the distributional assumption of the methadone doses in Section 5.1.

**Figure 3:**
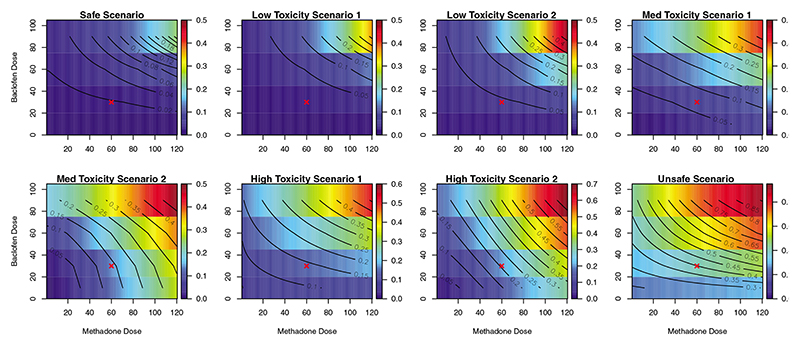
Contours of the combination toxicity scenarios explored in simulation study for various doses of methadone (*x*-axis) and baclofen (*y*-axis) generated from the five-parameter logistic model. The red cross corresponds to the 60 mg methadone and 30 mg baclofen—the combination used in the criterion to check whether it is clinically meaningful to continue to next phase given in Equation (3)

As continuous doses of methadone are considered, we parameterise the different scenarios evaluated in terms of a model and start from the model used by the design given in equation (1). Subsequently, we will generate matching scenarios using a competing model in Section 4.5. We study the robustness of the proposed design under these models and subsequently explore data simulated from a more flexible parametric model in Section 5.2. The contour lines (the lines of equal DLT risks) of the considered scenarios generated using the five-parameter logistic model are given in Figure 3.

The first scenario (top left corner) corresponds to the safe scenario with the highest dose of baclofen being in the target interval for a dose of methadone as high as 120 mg. The Low Toxicity and Med Toxicity scenarios are obtained by achieving 25% DLT risk for the highest dose of baclofen and 100, 80, 60, and 40 mg of methadone, respectively. The high toxicity scenarios correspond to the cases when the combination of 60 mg methadone and 30 mg baclofen is just below the target interval or just in the middle (20%). This combination is selected as this combination was chosen for the criterion that it is safe and clinically meaningful to proceed to the subsequent proof-of-concept study. Additionally, various interaction mechanisms and the dose−toxicity relationships are included for the completeness of evaluation— this can be seen from the various shapes of the contour lines. Note that the Medium Toxicity Scenario 2 corresponds to the case with almost no interaction between drugs—the interaction parameter is close to zero and equals to 0.10 − see Supporting Information for the exact values used to generate the scenarios. The last scenario (bottom right corner) corresponds to a highly unsafe scenario—this scenario will reveal with what probability the trial will be stopped early for toxicity in the unlikely event of highly toxic combinations. In each scenario, 2000 simulated trials with the sample size of up to *N* = 48 were generated.

### 4.4 Prior calibration

As discussed above, the proposed approach requires prior distributions to be defined for the model parameters to start the trial. The prior distributions to be used were calibrated over a number of combination−toxicity scenarios using a grid of various hyper-parameters of *μ*_0_,*μ*_1*i*_,*σ*_0*i*_,*σ*_1*i*_,*σ*_01,*i*_ (*i*=1,2) and *σ_η_* to yield good performance across a range of scenarios. Specifically, we have chosen the qualitative different scenarios: Low Toxicity Scenario 1 with the higher doses of the compound being safe or just above the upper toxicity bound; Medium Toxicity Scenario 1 with the target contour being in the middle of the combination toxicity grid; and High Toxic Scenario 2 with many combinations being unsafe. Then the set of hyper-parameters that result in the highest geometric average of accuracy was selected for the subsequent study.^19^ The ensuing values of the parameters were tried: *μ*_0*i*_={−4.00,−3.75,−3.50,−3.25,−3.00}, *μ*_1*i*_={0.0,0.05,0.15,0.30,0.40,0.50},*σ*_0*i*_={0.40,0.50,0.60,0.70,0.80},*σ*_1*i*_={0.15,0.25,0.35,0.45,0.55},*σ*_01,*i*_={−0.10,0.00,0.10}, *σ*_*η*_={0.75,1.00,1.25,1.50,2.00,5.00}. The values of hyperparameters given in Section 3.3 were found to yield the best performance in terms of the geometric mean of the average proportion of correct target dose recommendation.

### 4.5 Competing approach

We compare the performance of the proposed design to an extension of the model proposed by Bailey et al.^12^ that models various levels of one compound defined through a number of binary covariates. Specifically, the following model is used

$$\text{logit}\left(p\left(M,B_{k}\right)\right)=\alpha_{01}+\alpha_{11}\times \log\left(M/M_{*}\right)+\beta_{1}\times I\left(B_{k}\geq 30\right))+\beta_{2}\times I\left(B_{k}\geq 60\right))+\beta_{3}\times I\left(B_{k}\geq 90\right)),$$

where, as before, *α*_01_ and *α*_11_ are the intercept and slope parameters, I(·) is an indicator function. Note that the first two terms correspond to the single-agent methadone dose−toxicity model. It is easy to see that this parameterisation accounts for the monotonically increasing risk of toxicity with increasing dose of baclofen but does not model the baclofen−toxicity relationship directly. The rest of the design proceeds as in Section 2.3 with the only difference being the combination−toxicity model used to compute the probabilities of being safe and being in the target range. We will refer to this model as the design of Bailey et al.

The prior distributions for the parameters of the Bailey et al. design have been calibrated to match the prior distribution of the risk of toxicities induced by the proposed design at particular combinations to ensure a fair comparison. Specifically, we fixed the dose of methadone at *M* = 60mg (the dose used in the stopping early for safety criterion) and then chose values of parameters that provided approximately the same mean and variance of the risk distribution at combination with 30, 60, and 90 mg of baclofen. The hyper-parameter corresponding to the intercept and slope are taken from the proposed design as this part of the model is unchanged, and the parameters corresponding to binary covariates have prior distribution $\left(\log\left(\beta_{1}\right),\log\left(\beta_{2}\right),\log\left(\beta_{3}\right)\right)∼𝒩\left(\left(-1.0,-1.2,-1.2\right),\tilde{\sum }\right)$ with

$$\tilde{\sum }=\left[\begin{array}{ccc} 0.70^{2} & 0 & 0\\ 0 & 0.875^{2} & 0\\ 0 & 0 & 1.10^{2} \end{array}\right].$$

As the scenarios in Section 4.3 are generated from the proposed model, for a fairer comparison of the two approaches we have also included the matching scenarios generated from the Bailey et al. model.^12^ These scenarios were specified by approximately matching the toxicity rate at the safety−check combination (60 mg methadone, 30 mg baclofen) and 90 mg of baclofen and 120 mg methadone at the scenarios considered in the main body of the manuscript. The contour plots of these scenarios are given in Supporting Information. We will mark the scenarios generated from the proposed model by “2BLRM” standing for the two-dimensional Bayesian Logistic Regression Model, and the scenarios generated from the Bailey et al. model by “BLRM−Covariate.”

### 4.6 Numerical results

The operating characteristics of the proposed design and the comparator by Bailey et al. based on 2000 simulated trials for each of the combination−toxicity scenarios are given in Table 3.

Considering the performance of the proposed design under scenarios generated from the five-parameter logistic model (2BLRM), if the trial was not stopped early, the average proportion of patients in the next testing phase (proof-of-concept study) that have been correctly allocated to their target dose of baclofen is above 60%. The best performance can be seen under the Safe and Low Toxicity 1 scenarios—more than 90% of patients on average are correctly allocated (93.4% and 91.3%, respectively) and almost all of them are assigned a safe combination (99.9% and 99.0%, respectively). In more toxic scenarios, the average proportion of correct selection in the subsequent proof-of-concept study varies between 61% and 78%. It is noteworthy that, even if the design selects a combination that is not in the target interval (15%−25% toxicity), it is highly likely that the selected combination is safe for that patient. Specifically, the average proportion of patients allocated to safe doses ranges from 86.1% to 96.8% under low, moderate, and High Toxicity 1 scenarios and is slightly lower, 74.0% under High Toxicity Scenario 2.

Concerning the ethical consideration of the trial conduct, the average proportion of DLTs experienced in the trial is either well below the target interval or lies within it, in all scenarios except unsafe scenarios. Higher proportions of DLTs only happen in more toxic scenarios, namely Median 2 and High 1−2 where many combination are unsafe. In order to explore the combination−toxicity relationship, some patients may be assigned to unsafe combinations and by this we learn that these combinations are indeed unsafe. This reflects a well-known trade-off between the accuracy of a dose-escalation design and ethical constraints.^20^ Reassuringly, the proportion of safe selections by the end of trial is high and ranges between 74% and nearly 90%. Finally, under the unsafe scenario with all combinations being highly toxic, the trial is stopped with probability 88.5%.

Comparing the proposed design to the model with binary covariates under scenarios generated from the fiveparameter logistic model, it was found that the proposed design outperforms the alternative in all safe scenarios with the difference in the average proportion of correct recommendations ranging between 2% and 13% (the mean difference of 8%). Furthermore, the proposed design resulted in a higher proportion of safe selections in all safe scenarios. The only scenario generated from the five-parameter logistic model, under which Bailey et al. outperformed the proposed design is the Unsafe scenario: the proportion of terminations is 10% higher and it requires nearly 10 fewer patients to reach the conclusion.

The comparison under scenarios generated from the Bailey et al. model with covariates is in line with the above findings but with smaller differences. It was found that the proposed model resulted in a higher proportion of correct selections, on average, with an average difference of 4.2%. However, the proposed model is slightly outperformed (with difference up to 3.3%) under two scenarios: Low Toxicity 2 and High Toxicity 1.

Overall, the proposed design is able to find the target combinations with high probability in many different scenarios, and was found to allocate the majority of patients to safe doses. The design can also terminate the trial with high probability when toxicity risk is high.

## 5 Robustness

### 5.1 Random combination-toxicity scenarios

While the simulation study above covers qualitatively different scenarios, they only cover eight potential scenarios. To study the operating characteristics of the design in more detail and under a large number of scenarios, we adopt the approach proposed by Clertant and O’Quigley^21^ and generated 1000 random combination−toxicity scenarios. Specifically, using the combination−toxicity model (1), the parameters of the model were randomly drawn from $\alpha_{01}∼𝒰\left(-5.00,-0.75\right),\alpha_{02}∼𝒰\left(-6.00,-0.75\right),\alpha_{11}∼𝒰\left(0.40,3.00\right),\alpha_{12}∼𝒰\left(0.10,2.00\right),\eta ∼𝒰\left(0.0,1.1\right).$ This generates a number of different dose combination toxicity scenarios (1000) under which the performance of the design will be studied. The robustness of the design under 1000 random scenarios generated under an alternative model is given in Section 5.3.

As there are many underlying combination−toxicity scenarios resulting from a random draw of the model parameters, we classify them according to the toxicity risk at the combination 60 mg of the *M* compound and 30 mg of the *B* compound (referred to as the “safety-check” combination) that is used in the safety criterion and the criterion to proceed to the next phase of the trial, and present the aggregated results for scenarios depending on different toxicity risk at this combination.

Under safe scenarios, we present the average proportion of patients correctly allocated to the target doses in all scenarios with the risk of toxicity at the safety-check combination being below some value *x*%. For unsafe scenarios, we present the mean proportion of trial terminations over all scenarios with the risk of toxicity at the safety−check combination being above some value *x*%.

**Figure 4:**
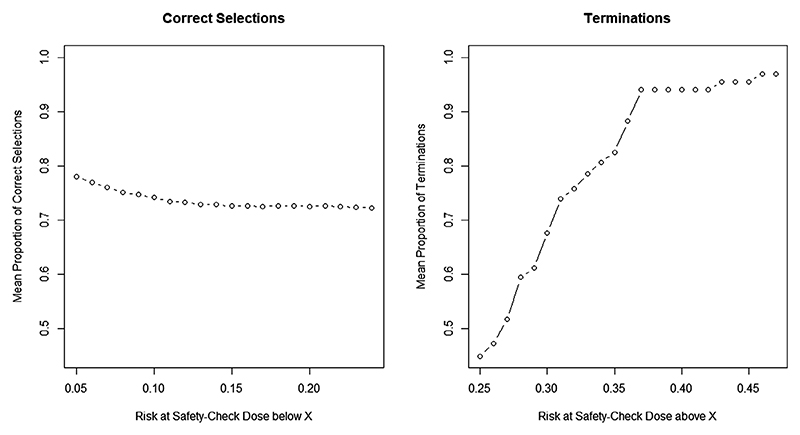
Results for 1000 random scenarios generated from the proposed logistic model. Left panel: Average proportion of correctly allocated patients in safe scenarios with the risk of toxicity at 60 mg methadone, 30 mg baclofen (referred to as safety-check combination) below *x*% given on the *x*-axis; Right panel: Mean proportion of trial terminations over unsafe scenarios with the risk of toxicity at the safety-check combination being above *x*% given on the *x*-axis

The summary of operating characteristics with 1000 randomly generated scenarios (and performance of each is assessed in 1000 simulations) is given in Figure 4.

It can be seen that the proposed design has better performance, around 80%, in the scenarios with the safety−check combination being associated with the risk of toxicity below 5%. Although this probability decreases slightly as the toxicity risk increases, the average performance in all safe scenarios is around 72% and the design is robust in scenarios with various combination−toxicity relationships. For the unsafe scenarios, the proportion of trials terminated early for safety reasons increases as the probability of toxicity at the safety−check combination increases as expected. In scenarios with toxicity risk 10% above the upper bound of the target interval (15%−25%), the proportion of termination is above 90%.

The findings above confirm the results under the eight fixed scenarios that the proposed design can find the optimal dose of baclofen robustly with high probability under a range of scenarios. We study the robustness of the design under model misspecification and alternative distributional assumptions of the doses of *M* below.

### 5.2 Heavy-tailed distributions of externally defined compound

In Section 3, the accuracy characteristics of the design were evaluated in terms of future patients in a subsequent study. In the context of the motivating trial, this translated into the proportion of patients (out of 112) that received the target dose. The underlying assumption when generating the methadone dose for each of these patients was that they come from the same patient population as those in the dose-finding trial. In general, however, the patients in the next stages may have different characteristics, for example, due to different eligibility criteria. We therefore also evaluate whether the design can predict the target combination for the patient population when the distribution of the patient-specific methadone doses is different from the distribution used in the dose-finding study.

To study the impact of this, we consider the following distributional assumption for the methadone doses for the 112 patients in the subsequent trial. The methadone doses in the dose-finding study still follow the truncated normal distribution as in Section 3. However, when evaluating performance in the subsequent hypothetical patients, their methadone doses have.

1. Non-standard Student’s distribution with *ν* = 10 degrees of freedom with the same mean and scale parameter as the normal distribution in the dose-finding study but with heavier tails of the distribution.
2. The uniform distribution U(1,150)—where 1 and 150 mg and the hypothetical lowest and the highest doses of methadone eligible for further consideration.

Although, the historical data from the addiction services at Central North West London NHS foundation trust show that the distribution of the prescribed methadone doses in the patients’ population of interest has a bell shape, with the latter distribution assumption, we explore to what extent the proposed design is robust to the methadone doses distribution.

The summary of operating characteristics based on 2000 simulated trials and with the methadone for 112 patients having either a truncated non-standard Student’s or uniform distribution are given in Table 4.

Considering the case of Student’s t-distribution for methadone doses, under scenarios with low toxicity, the prediction of the doses for the patients in the subsequent study is slightly improved (6%−10%) compared to the original case as now more patients receive methadone doses close to the bound (corresponding to a heavier tails of the distribution) which are safe under these scenarios. However, under the medium and highly toxic scenarios, the performance is worsened by 4%−10%. At the same time, the design is still able to find the correct dose with high accuracy and the proportion of safe recommendation is still higher. It is 70% in High Toxic Scenario 2 and is above 80% in the rest of the scenarios.

Considering the case of uniformly distributed methadone doses, the design is able to make accurate baclofen dose recommendations under a range of scenarios but with lower probabilities compared to both the Normal and Student’s cases: the average proportion of correct selections varies between nearly 48% and 77% (compared to 55%−99% under the Student’s *t*-distribution). Reassuringly, the proportion of safe dose recommendations is still high in all scenarios, 91%−95% under six out of seven scenarios with safe combinations, and 65% in the High Toxicity Scenario 2.

Importantly, in the unsafe scenarios, the performance of design is unchanged and the trial stops early for safety with high probability, although the proportion DLT responses are higher.

Overall, the average design performance is robust to the distributional assumption for M and the design can lead to reliable combination recommendation even if the patients in the next studies have the distribution of externally defined doses different from the corresponding distribution in the dose-finding trial.

### 5.3 Model misspecification

Previously we have assumed that both the data and the model assume the same parametric form for the combination− toxicity relationship. However, in practice, the true combination−toxicity curve may be different. In this section, we consider an alternative parametric form to generate the combination−toxicity. Specifically, we will consider a more flexible seven-parameter Bliss model on the DLT-probabilities^22,23^ given by the following form:

$$p\left(M_{j},B_{k}\right)=\beta_{2}+\left(\beta_{1}-\beta_{2}\right)\left(1-\frac{1}{{\left({\left(1+M_{j}/\left(M_{*}\beta_{3}\right)\right)}^{\beta_{5}}{\left(1+B_{k}/\left(B_{*}\beta_{4}\right)\right)}^{\beta_{6}}\right)}^{\beta_{7}}}\right)$$

and restrict parameters such that the probabilities lies between 0 and 1, where *M*_⁎_ and *B*_⁎_ are reference doses as before. Similar to the approach above, we randomly generate 1000 various combination−toxicity scenarios by drawing the coefficients from the following uniform distributions $\beta_{1}∼𝒰\left(0.0,0.4\right),\;\beta_{2}∼𝒰\left(0.0,0.25\right),\;\beta_{3},\beta_{4},\beta_{5},\beta_{6}∼𝒰\left(0.5,1.5\right),\beta_{7}∼𝒰\left(0.75,1.5\right).$ This model is used to generate the toxicity outcome in both the dose-finding stage of the trial and for the evaluation of the predictive properties of the design in the subsequent study but not to make dose-escalation decisions. As before, we summarise the results in terms of the toxicity at the safety−check combination.

**Figure 5:**
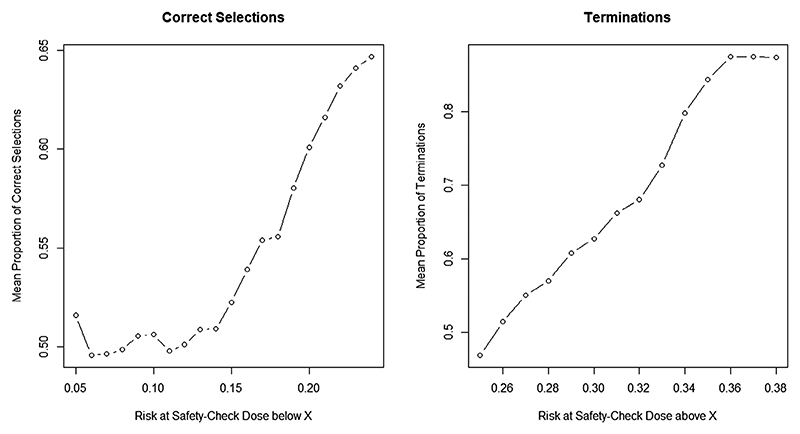
Results for 1000 random scenarios generated from the Bliss model. Left Panel: Average proportion of correctly allocated patients in safe scenarios with the risk of toxicity at 60 mg methadone, 30 mg baclofen (safety-check combination) below *x*% given on the *x*-axis; Right panel: Mean proportion of trial terminations over unsafe scenarios with the risk of toxicity at the safety-check combination being above *x*% given on the *x*-axis

The summary of operating characteristics in 1000 randomly generated scenarios (and performance of each is assessed in 1000 simulations) under the Bliss model for the toxicity generating is given in Figure 5.

In less toxic scenarios, in which the toxicity risk at the safety−check dose is between 5% and 15%, the average accuracy is around 50% and increases up to 65% in more toxic scenarios when the reference dose is in the target range. Therefore, the proposed BLRM model is more accurate in more toxic scenarios. Importantly, the proportion of the safe selection was between 97% and 99% under all considered scenarios (not shown).

Concerning the proportion of terminations in unsafe scenarios, similarly to the previous results, when the risk at the safety−check combination is 35% or above, the proportion of terminations is just under 90%. Therefore, the design can stop the trial earlier even under the model misspecification if the combination is excessively toxic.

Overall, while the violations of the model assumption has been found to lead to slightly worsen the design characteristics in terms of accuracy, the design can still identify the right dose with the probability at least 50%, on average, in many considered scenarios. It also recommends the safe combinations with probability of at least 70% even under the most toxic scenarios.

## 6 Discussion

A model-based design for a dual-agent combination finding study with patient-specific dose recommendations was proposed in this work. The two distinguishing features of the considered setting is that the (continuous) dose of one compound is defined externally, and the objective of the trial is to define the *dosing function* that would recommend a safe combination of another compound given the externally defined dose. A two-parameter BLRM that was previously used in conventional combination studies was extended to tackle these problems. It is found that the proposed design can provide accurate patient-specific combination recommendations and is robust to the model misspecification and various distributional assumptions of the (continuous) externally defined doses.

To justify the trial design and sample size to funders we proposed metrics that quantify and demonstrate the accuracy of design based on its predictive properties for the patients to be enrolled in the next phase. Specifically, the predictive proportion of patients in the subsequent testing phase that will receive (i) their target combination, and (ii) receive a combination that is safe for them. Such metrics could also be used to justify trial designs in other dual agent trials with non-patient specific doses. If its currently unknown how many patients will be enrolled in the next phase, the performance of the design in terms of its predictive properties for a sample size based on a contextually appropriate standardized effect could be considered within evaluations. For example, predictive properties for a sample size to attain 90% power to detect a medium effect size (0.5 *SD*) with two-sided 5% significance, could be explored. We also demonstrated how combination escalation/de-escalation paths can clearly provide the team and funders with reassurances that the decisions taken by the proposed design are in line with expectations.

As the goal of the trial is to define the dosing function (rather than a single combination) the proposed parametric model can (and should) be used to define the safe combination in subsequent study. As the patient-specific recommendation is a cornerstone of the considered clinical setting, carrying over just a single combination recommendation from the Phase I study would inevitably result in the loss of information and administering suboptimal (or simply harmful for some patients) combinations in subsequent phases. The model-based nature of the design also allows for incorporation of new information obtained in the subsequent study for a more accurate estimation of the dosing function.

While demonstrating good operating characteristics of a design is important to ensure an accurate performance regardless of the underlying scenario, it is also crucial that the design is clearly communicated and is not considered as a “black box” by the clinical team. While decision trees were found to be quite useful to accomplish this goal,^24^ these can become quite cumbersome in the combination setting with externally defined (and continuous) doses of one compound. To approach this, we have proposed to demonstrate several escalation trajectories of how the trial would proceed under the proposed design using the underlying model. In this paper, we have shown two possible dose-escalation paths. These trajectories were used for iterative discussions with the team to inform the choice of the prior distribution and ensuring that the proposed model-based design leads to the intuitive decisions making. Specifically, these were used to check that the model is not overly aggressive (too quick in escalation or early stopping) or is not overly conservative—the doses are indeed escalated with the combination that has proven to be safe. In general, we suggest that demonstrating the individual trial behaviour (in an appropriate for the trial’s setting manner) should be one of the parts of the design evaluation, on the top of a conventionally performed simulation study. If the demonstrated escalation/de-escalation decisions would raise any doubts in the clinical team, a refining prior parameters might be required.

The proposed BLRM underlying the presented trial design can be adopted and applied more generally in alternative clinical settings testing dual agents in combination with patient specific levels of one agent (be these discrete or continuous). The flexibility of the model-based approach can accommodate various group sizes, a different target toxicity interval, alternative safety/stopping constraints, and skipping the doses (if deemed clinically acceptable).

Finally, the design evaluated in this manuscript uses calibrated values for the prior distributions of the parameters of the model, the values resulting in good operating characteristics of the design across many different scenarios. This strategy was chosen assuming limited knowledge about the toxicity of the combination of the compounds under investigation. At the same time, there might be some historical information from previous trials on each compound individually. Moreover, the clinical team can provide information on their expectations about the toxicity of combinations given their clinical knowledge of the mechanism of action of the compounds. While there are methods on how this information can be used in Phase I clinical trials and how it affects the operating characteristics in terms of the conventional single dose/combination selection,^6,14,25^ further research on the benefits these (or similar methods) can provide in estimating the dosing function in Phase I studies will be conducted.

## Supplementary Material

Supplementary file

## Figures and Tables

**Table 1:** Example of the dose-escalation path if no DLTs are observed in the trial

Group	M doses	B doses	DLTs	ℙ(unsafe)	ℙ(safe)	*B* = 10mg	*B* = 30mg	*B* = 60mg	*B* = 90mg
1	31, 47, 86	10, 10, 10	0, 0, 0	0%	54%	(147,150)	(105,146)	(73,104)	(10,72)
2	66, 29, 44	30, 30, 30	0, 0, 0	0%	56%	(148,150)	(107,147)	(77,106)	(10,76)
3	28, 56, 66	60, 60, 60	0, 0, 0	0%	62%	−	(116,150)	(83,115)	(10,82)
4	18, 84, 83	90, 90, 90	0, 0, 0	0%	76%	−	(137,150)	(106,136)	(10,105)
5	74, 30, 75	90, 90, 90	0, 0, 0	0%	83%	−	(143,150)	(118,144)	(10,117)
6	82, 90, 10	90, 90, 90	0, 0, 0	0%	88%	−	(147,150)	(130,146)	(10,129)
7	64, 60, 91	90, 90, 90	0, 0, 0	0%	93%	−	−	(137,150)	(10,136)

*Note:* M doses are the externally prescribed fixed patient-specific doses of methadone; B doses are the baclofen doses recommended by the design; ℙ(unsafe) and ℙ(safe) are the probabilities in Equations (3) and (4), respectively, used for early stopping; columns *B* = XXmg correspond to the intervals of methadone that a patient should be prescribed in order to be recommended dose XXmg of baclofen.

**Table 2:** Example of the dose-escalation path if DLTs are observed in the trial

Group	M doses	B doses	DLTs	ℙ(unsafe)	ℙ(safe)	*B* = 10mg	*B* = 30mg	*B* = 60mg	*B* = 90mg
1	29, 85, 44	10, 10, 10	0, 0, 0	0%	54%	(146,150)	(105,145)	(74,104)	(10,73)
2	20, 71, 53	30, 30, 30	0, 0, 0	0%	57%	(150,150)	(108,149)	(76,107)	(10,75)
3	37, 18, 22	60, 60, 60	0, 0, 0	0%	58%	−	(111,150)	(79,110)	(10,78)
4	58, 47, 72	90, 90, 90	1, 0, 0	0%	32%	(144,150)	(101,143)	(60,100)	(10,59)
5	45, 99, 66	90, 60, 60	0, 0, 0	0%	42%	−	(112,150)	(69,111)	(10,68)
6	38, 70, 61	90, 60, 90	0, 0, 0	0%	51%	−	(128,150)	(79,127)	(10,80)
7	75, 83, 36	90, 60, 90	0, 0, 0	0%	60%	−	(138,150)	(101,137)	(10,100)
8	69, 56, 38	90, 90, 90	1, 0, 0	0%	33%	−	(112,150)	(77,111)	(10,76)
9	48, 34, 67	90, 90, 90	1, 0, 0	0%	21%	−	(102,150)	(63,101)	(10,64)
10	11, 68, 52	90, 60, 90	0, 0, 1	0%	11%	(145,150)	(92,144)	(60,91)	(10,59)
11	47, 29, 79	90, 90, 60	0, 0, 0	0%	14%	−	(97,150)	(59,96)	(10,58)
12	54, 80, 59	90, 60, 60	0, 1, 1	0%	4%	(128,150)	(82,127)	(46,81)	(10,45)
13	57, 48, 59	60, 60, 60	0, 0, 0	0%	5%	(132,150)	(85,131)	(56,84)	(10,55)
14	59, 20, 10	60, 90, 90	0, 0, 0	0%	5%	(133,150)	(86,132)	(57,85)	(10,56)
15	44, 76, 79	90, 60, 60	0, 0, 0	0%	7%	(139,150)	(90,138)	(60,89)	(10,59)
16	66, 19, 54	60, 90, 90	0, 0, 0	0%	8%	(143,150)	(93,142)	(63,92)	(10,62)

*Note:* M doses are the externally prescribed doses of methadone; B doses are the baclofen doses recommended by the design; ℙ(unsafe) and ℙ(safe) are the probabilities in Equations (3) and (4), respectively, used for early stopping; columns *B* = XXmg correspond to the intervals of methadone that a patient should be prescribed in order to be recommended dose XXmg of baclofen.

**Table 3:** Operating characteristics of the proposed design and the design by Bailey et al. with binary covariates in the considered scenarios generated from the five-parameter logistic model (2BLRM) and Bailey et al. (BLRM−covariate)

	Scenario generated from	% of correct selections	% of safe selections	Proportion of DLTs	Average sample size	Stopped unsafe	Stopped safe
Safe scenario							
Proposed	2BLRM	93.4%	99.9%	6.1%	44.3	0.0%	22.5%
Bailey et al.		80.3%	99.9%	6.2%	48.0	0.0%	0.0%
Proposed	BLRM-Covariate	62.9%	100.0%	13.9%	47.7	0.0%	1.6%
Bailey et al.		51.8%	100.0%	13.0%	48.0	0.0%	0.0%
Low Toxicity 1							
scenario							
Proposed	2BLRM	91.3%	99.0%	7.4%	45.8	0.0%	13.5%
Bailey et al.		79.5%	98.4%	7.7%	48.0	0.0%	0.0%
Proposed	BLRM-Covariate	70.8%	79.9%	15.8%	47.9	0.0%	0.1%
Bailey et al.		63.0%	77.8%	13.6%	48.0	0.0%	0.0%
Low Toxicity 2							
scenario							
Proposed	2BLRM	77.8%	96.8%	10.6%	47.3	0.0%	3.4%
Bailey et al.		75.4%	95.1%	10.8%	48.0	0.0%	0.0%
Proposed	BLRM-Covariate	51.3%	80.3%	17.4%	48.0	0.0%	0.0%
Bailey et al.		54.6%	87.6%	16.4%	47.9	0.3%	0.0%
Medium Toxicity 1							
1 scenario							
Proposed	2BLRM	61.5%	95.1%	14.6%	47.9	0.0%	0.1%
Bailey et al.		58.9%	93.2%	14.7%	47.7	0.8%	0.0%
Proposed	BLRM-Covariate	68.9%	93.2%	14.7%	48.0	0.0	0.0%
Bailey et al.		64.9%	88.9%	15.4%	47.6	1.2%	0.0%
Medium Toxicity 2							
scenario							
Proposed	2BLRM	74.1%	86.1%	18.1%	47.8	1.1%	0.0%
Bailey et al.		64.0%	79.8%	18.1%	46.5	6.1%	0.0%
Proposed	BLRM-Covariate	78.6%	89.4%	15.4%	47.7	0.9%	0.0%
Bailey et al.		72.3%	80.2%	15.9%	46.6	4.9%	0.0%
High Toxicity 1							
scenario							
Proposed	2BLRM	69.0%	89.3%	18.2%	47.9	0.1%	0.0%
Bailey et al.		62.5%	79.8%	18.4%	45.9	8.6%	0.0%
Proposed	BLRM-Covariate	55.3%	61.6%	20.9%	47.9	0.9%	0.0%
Bailey et al.		57.8%	63.2%	20.4%	43.3	19.1%	0.0%
High Toxicity 2							
scenario							
Proposed	2BLRM	74.0%	74.0%	21.5%	47.0	6.8%	0.0%
Bailey et al.		62.3%	62.3%	22.0%	40.9	31.9%	0.0%
Proposed	BLRM-Covariate	61.4%	72.4%	17.7%	47.4	9.4%	0.0%
Bailey et al.		56.5%	67.2%	18.9%	42.4	22.1%	0.0%
Unsafe scenario							
Proposed	2BLRM	-	-	29.4%	29.4	88.5%	0.0%
Bailey et al.		-	-	34.9%	19.3	98.2%	0.0%

*Note:* Results are based on 2000 replicated trials for each scenario.

**Table 4:** Operating characteristics of the proposed design under Student’s distribution and uniform on the interval (1,150) of the methadone doses in eight considered scenarios

	Correct selections	Safe selections	Proportion of DLTs	Average sample size	Stopped unsafe	Stopped safe
Safe scenario						
Student’s *t*-distribution	99.0%	100.0%	5.9%	44.1	0.0%	23.0%
Uniform distribution	77.0%	95.7%	6.1%	44.1	0.0%	23.3%
Low Toxicity Scenario 1						
Student’s *t*-distribution	98.1%	100.0%	7.5%	45.9	0.0%	12.7%
Uniform distribution	68.2%	91.5%	7.5%	46.2	0.0%	10.1%
Low Toxicity Scenario 2						
Student’s *t*-distribution	87.3%	99.9%	10.6%	47.4	0.0%	3.6%
Uniform distribution	58.3%	91.4%	10.5%	47.5	0.0%	3.1%
Medium Toxicity Scenario 1						
Student’s *t*-distribution	55.7%	98.9%	14.7%	47.9	0.0%	0.5%
Uniform distribution	47.8%	96.6%	14.6%	47.9	0	0.3%
Medium Toxicity Scenario 2						
Student’s *t*-distribution	64.0%	82.9%	18.1%	47.8	1.2%	0.0%
Uniform distribution	62.9%	81.1%	18.0%	47.9	0.8%	0.0%
High Toxicity Scenario 1						
Student’s *t*-distribution	60.6%	82.9%	18.3%	47.9	0.1%	0.0%
Uniform distribution	57.2%	93.5%	18.2%	47.9	0.5%	0.0%
High Toxicity Scenario 2						
Student’s *t*-distribution	70.3%	70.3%	21.5%	46.9	6.6%	0.0%
Uniform distribution	65.1%	65.1%	21.4%	46.8	7.1%	0.0%
Unsafe scenario						
Student’s *t*-distribution	-	-	35.1%	29.1	88.8%	0.0%
Uniform distribution	-	-	33.2%	29.2	88.6%	0.0%

*Note*: Results are based on 2000 replicated trials in each scenario.

## Data Availability

Software in the form of R code implementing the proposed design is available on GitHub (https://github.com/dose-finding/2blrm-detox-trial).
