# Supplementary material for: A dose-finding design for dual-agent trials with patient-specific doses for one agent with application to an opiate detoxification trial
Source: Pharm Stat. Author manuscript; Available in PMC 2022 Apr 11. (PMC7612599; doi:10.1002/pst.2181)
Supplement: Supplementary file [file EMS144019-supplement-Supplementary_file.pdf]

Received: Added at production | Revised: Added at production | Accepted: Added at production
DOI: xxx/xxxx

ARTICLE TYPE

A dose-finding design for dual-agent trials with patient-specific doses for one agent with application to an opiate detoxification trial. Supplementary Materials.

Pavel Mozgunov*[1] | Suzie Cro[2] | Anne Lingford-Hughes[3] | Louise M Paterson[3] | Thomas Jaki[1,4]

[1]MRC Biostatistics Unit, University of Cambridge, UK
[2]Imperial Clinical Trials Unit, School of Public Health, Imperial College, UK
[3]Division of Psychiatry, Department of Brain Sciences, Imperial College, UK
[4]Department of Mathematics and Statistics, Lancaster University, UK

Correspondence
*Pavel Mozgunov, Email: pavel.mozgunov@mrc-bsu.cam.ac.uk

Summary

KEYWORDS:
Baclofen, Combination Trial, Dose-Finding, Dose Individualisation, Methadone, Opiate Detoxification

1 | PARAMETERS FOR CONSIDERED SCENARIOS

The parameters of the 5-parameter logistic model used in the simulation study in Section 4 and to plot the contours of equal DLT rates (Figure 3, Section 4.3) are given in Table 1.

TABLE 1 The parameters of the 5-parameter logistic model used in the simulation study in Section 4.

Scenario	α_{01}	α_{11}	α_{02}	α_{12}	η
Safe	-4.20	0.90	-5.45	0.05	0.85
Low Toxicity 1	-4.20	1.00	-5.3	0.20	1.00
Low Toxicity 2	-3.40	0.50	-5.60	0.50	1.00
Medium Toxicity 1	-2.50	1.50	-3.60	1.00	0.40
Medium Toxicity 2	-2.50	2.50	-2.00	1.50	0.10
High Toxicity 1	-2.50	1.00	-2.50	0.20	0.50
High Toxicity 2	-2.00	1.00	-2.00	1.50	0.40
Unsafe	-1.35	0.90	-1.35	0.00	0.85

2 | POSTERIOR DISTRIBUTIONS OF THE INTERACTION TERM IN THE PROPOSED MODEL

The primary target of the Phase I trial is to correctly estimate the recommended baclofen dose rather than the estimation of the parameters. Hence, the posterior estimates of the interaction parameter might not be close to the true value but the design can still recommend the correct combination with high probability (as it fits the curve in the neighbourhood of the correct doses correctly). Opting to a more flexible model is primarily motivated by being able to fit various combination-toxicity relationships and approximate it accurately, regardless of the location of the correct combination on the studied grid. At the same time, the interpretation of the multiplication should have an explanation and interpretation.

The distributions of the mean posterior estimates of the interaction parameter η in 7 safe scenarios (generated from the proposed 5-parameter logistic model) in Figure 1.

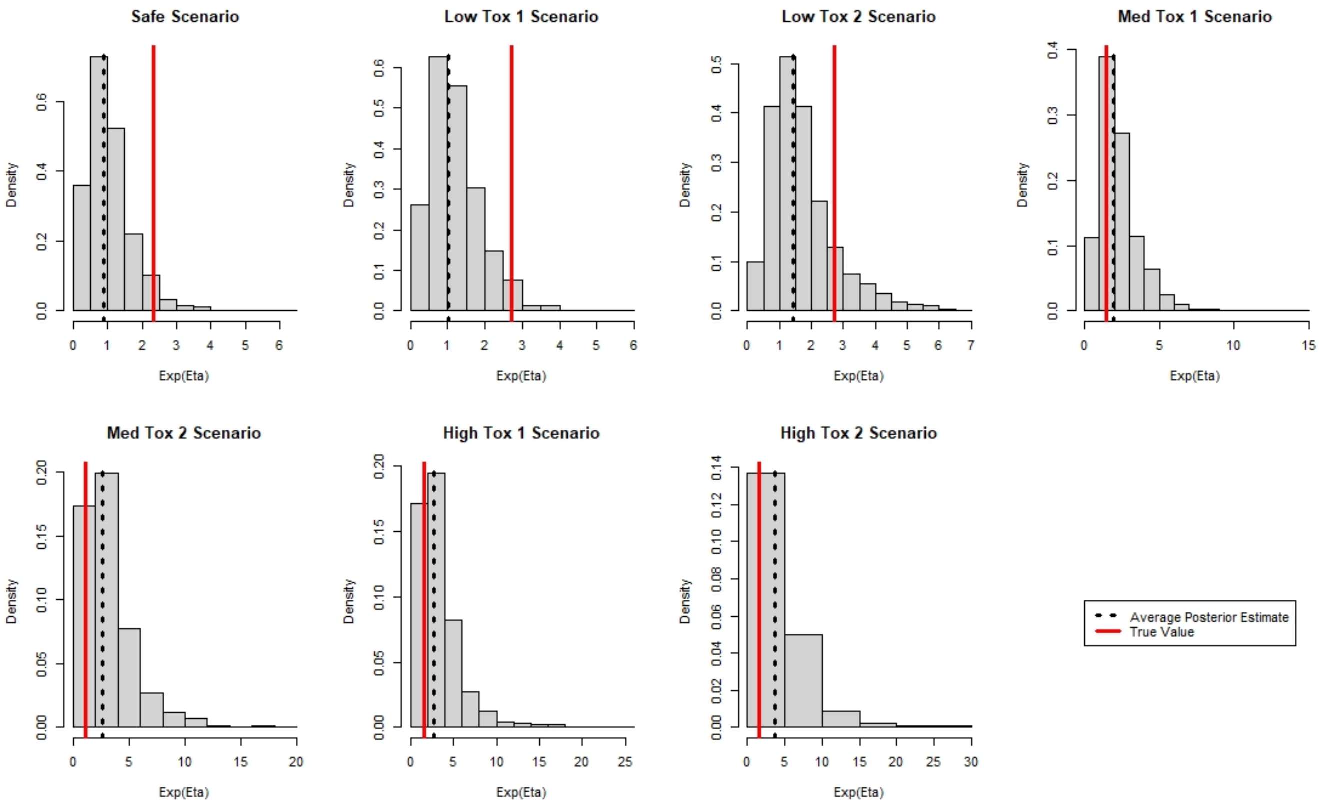

FIGURE 1 Distributions of the mean posterior estimates of the interaction parameter η in 7 safe scenarios. The dashed black line corresponds to the average taken over the mean posterior estimates, and the solid red line corresponds to the true value of the parameter under the given scenario.

From the distribution above, we see that 95% of all posterior means (across 2000 simulations) of the interaction multiplicator (on the odds scale) are below 2.3 (for safe and low toxicity scenarios) and are below 7.9 (under medium toxicity 2 and high toxicity scenarios). The means range from around 1 for the safe scenario to around 4 under the high toxicity scenarios 2. This suggests that the resulting multiplication model provides results on the sensible range of values.

3 | RESULTS FOR VARIOUS SAMPLE SIZES

The results in the main body of the paper concerned the sample size coming from the motivating trial - $N = 48$. Figure 2 presents the average proportions of patients correctly allocated in the subsequent study using various total sample sizes $N = 24, 30, 36, 42, 48$ under 7 scenarios considered in the main paper.

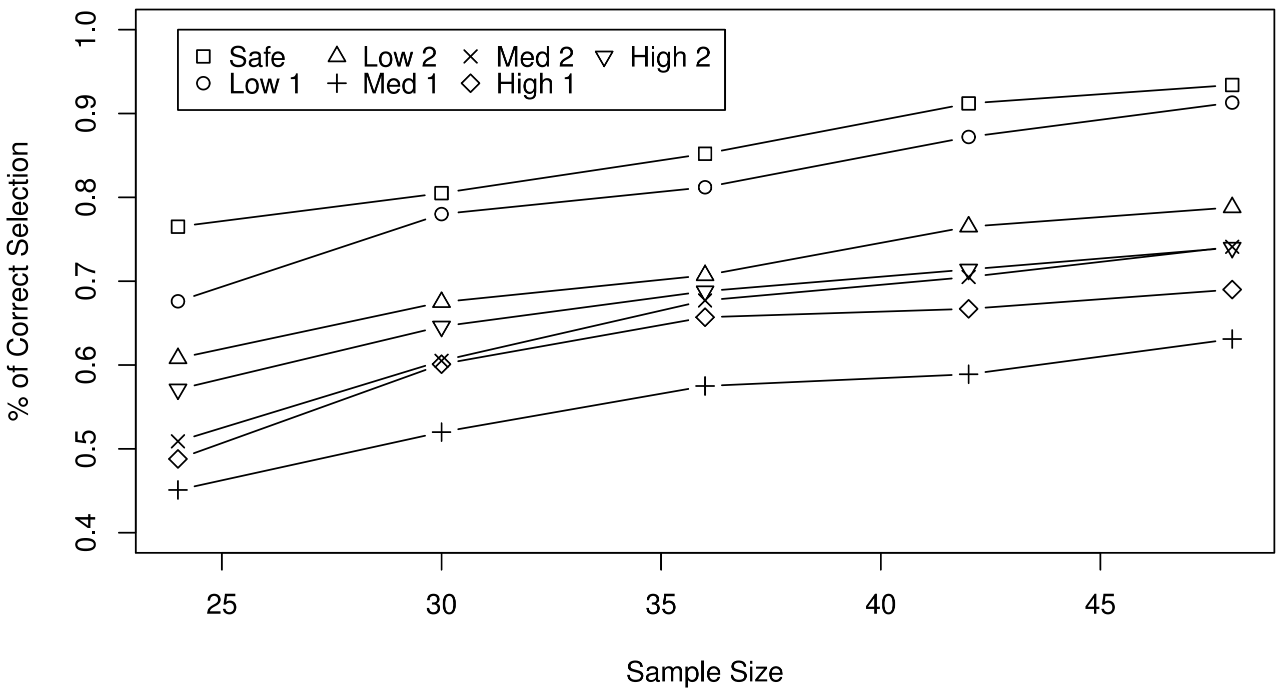

FIGURE 2 Average proportions of patients correctly allocated in the subsequent study using various total sample sizes $N = 24, 30, 36, 42, 48$.

Comparing the lowest and the highest sample sizes, the reduction in the average proportion is around 20% across all considered scenarios.

□
